# The Cooperative Revolution Reaches Clinical Psychology and Psychotherapy: An Example From Germany

**DOI:** 10.32872/cpe.4459

**Published:** 2021-03-10

**Authors:** Jürgen Margraf, Jürgen Hoyer, Thomas Fydrich, Tina In-Albon, Tania Lincoln, Wolfgang Lutz, Angelika Schlarb, Henning Schöttke, Ulrike Willutzki, Julia Velten

**Affiliations:** a Mental Health Research and Treatment Center, Ruhr University Bochum, Bochum, Germany; b Clinical Psychology and Psychotherapy, Technical University of Dresden, Dresden, Germany; c Department of Psychology, Humboldt-Universität zu Berlin, Berlin, Germany; d Clinical Child and Adolescent Psychology and Psychotherapy, University of Koblenz-Landau, Landau, Germany; e Clinical Psychology and Psychotherapy, Universität Hamburg, Hamburg, Germany; f Clinical Psychology and Psychotherapy, Trier University, Trier, Germany; g Clinic Psychology and Psychotherapy of Children and Adolescents, Bielefeld University, Bielefeld, Germany; h Clinical Psychology and Psychotherapy, Osnabrück University, Osnabrück, Germany; i Clinical Psychology and Psychotherapy, University Witten/Herdecke, Witten, Germany; Philipps-University of Marburg, Marburg, Germany

**Keywords:** psychotherapy research, outpatient clinics, collaborative research, replication crisis

## Abstract

**Background:**

Psychology is at the beginning of a cooperative revolution. Traditionally, psychological research has been conducted by individual labs, limiting its scope in clinical samples and promoting replication problems. Large-scale collaborations create new opportunities for highly powered studies in this resource-intensive research area. To present the current state of a Germany-wide platform for coordinating research across university outpatient clinics for psychotherapy.

**Method:**

Since 1999, over 50 such clinics were created in Germany. They represent a unique infrastructure for research, training, and clinical care. In 2013, a steering committee initiated a nationwide research platform for systematic coordination of research in these clinics (German abbreviation “KODAP”). Its main goal is to aggregate and analyze longitudinal treatment data – including patient, therapist, and treatment characteristics – across all participating clinics.

**Results:**

An initial survey (100% response rate) yielded recommendations for improved integration of data collection. Pilot data from 4,504 adult (16 clinics) and 568 child and adolescent patients (7 clinics) proved feasibility of data transfer and aggregation despite different data formats. Affective, neurotic, stress, and somatoform (adults) and anxiety and behavioral (children and adolescents) disorders were most frequent; comorbidity was high. Overcoming legal, methodological, and technical challenges, a common core assessment battery was developed, and data collection started in 2018. To date, 42 clinics have joined.

**Conclusions:**

KODAP shows that research collaboration across university outpatient clinics is feasible. Fulfilling the need for stronger cumulative and cooperative research in Clinical Psychology will contribute to better knowledge about mental health, a core challenge to modern societies.

## Abstract (highlights)

- Data from 4,504 adult and 568 child and adolescent patients were successfully aggregated across 23 outpatient clinics.
- Affective, neurotic, stress, and somatoform (adults) and anxiety and behavioral (children and adolescents) disorders were most frequent; comorbidity was high.
- Legal, methodological, and technical challenges were overcome, and a common core assessment battery was developed.
- 42 clinics have joined a Germany-wide research platform for systematic coordination of research in these clinics. Longitudinal data collection started in 2018.

Psychology and psychotherapy are at the beginning of a cooperative revolution (Chartier et al., 2018; Spellman, 2015). Traditionally, research in these fields has been conducted by individual labs, limiting its scope in clinical samples and promoting replication problems. In response to the so-called “replication crisis” in medicine, psychology and related fields (Camerer et al., 2018; Dumas-Mallet et al., 2017; Ioannidis, 2005; Open Science Collaboration, 2015; Pashler & Wagenmakers, 2012), the search for causes revealed methodological issues including insufficient sample sizes (Button et al., 2013; Flint et al., 2015; Rossi, 1990; Simmons et al., 2011) and the “file drawer problem” (aka publication bias; Kirsch et al., 2002; Rosenthal, 1979; Turner et al., 2008). These proximal causes are worsened by misaligned incentives in a context of dwindling research funding and increasing pressure to publish or perish (Margraf, 2015; Spellman, 2015). In addition, basic aspects of our academic cultures may serve as major contributors to the crisis by accelerating a race that, under the motto "winner takes all", favors fundamentally undesirable developments (Fang & Casadevall, 2012b). These “cultural” aspects include an exaggerated cult of originality (Fang & Casadevall, 2012a) and the “toothbrush problem” (Mischel, 2008): We tend to treat other peoples’ theories like toothbrushes — every decent person uses one but no self-respecting person wants to use anyone else’s. If getting and keeping your job and status requires achieving “originality” by not building on anyone else’s work, it may directly undermine the goal of building a cumulative science (Mischel, 2008). The conflict applies not only to theories but also to therapies: The field is full of overstated claims of originality and uniqueness, leading to ill-founded distinctions and misguided competition that impede fruitful cooperation. As a result of this “disconnect between what is good for scientists and what is good for science” (Nosek et al., 2012, p. 616) we have a situation, where “most published research findings are false” (Ioannidis, 2005) and “most clinical research is not useful” (Ioannidis, 2016).

We cannot, however, simply deplore external pressures and individual misconduct, we must also devote our critical attention to the cult of originality and priority and the overemphasis on individual contributions that underlie them. We need to pursue an academic community that works collectively, albeit competitively, to advance theory and therapy. This requires developing common shared tools and a more serious quest for robust, replicable and consequential findings (Mischel, 2009). The importance of teamwork in science has never been greater (Fang & Casadevall, 2012a). Teams increasingly dominate science and are contributing the highest-impact and most reliable research. Collaborations, consortia and networks are essential for tackling many of the most important challenges in psychotherapy and psychosomatics. Luckily, scientists in psychology and medicine recently have opened up much more to new forms of increased collaboration, allowing them to initiate projects at a scale previously unattained. Perhaps the most visible hallmark of the cooperative revolution has been the rapid increase in large-scale collaborations such as ManyLabs, ManyBabies, Open Science Collaboration, Psychological Science Accelerator, Registered Replication Reports, and StudySwap (Chartier et al., 2018). Our research questions as well as our often still inadequate measurement accuracy typically require very large samples (Margraf, 2015). Large joint projects and individual projects coordinated with them must complement each other, and the necessary infrastructure must be developed. This should create new opportunities for highly powered studies even in resource-intensive areas such as psychotherapy research.

The present article describes the example of an innovative approach to collaborative psychotherapy research from Germany (Hoyer et al., 2015; In-Albon et al., 2019; Velten et al., 2017, 2018). Since Germany established the legal basis for psychotherapy outpatient clinics at university departments of Clinical Psychology in 1999, over 50 such clinics devoted to research (i.e., research clinics) and to clinical training of psychotherapists (i.e., training clinics) were created. Each year, many thousand patients across all age and clinical groups are treated under routine clinical conditions as well as in circumscribed research projects (In-Albon et al., 2019; Velten et al., 2018). Together, they represent a unique infrastructure for research, training and clinical care that rapidly has proven to be an important facilitator of research in psychotherapy and mental health. The clinics routinely gather a large amount of data on therapy outcomes as well as on patient and therapist characteristics (Velten et al., 2017). High standards of quality assurance are achieved in these outpatient clinics through regular, standardized diagnostic assessments. These data can also be used for research, in particular psychotherapy research (e.g., Ziem & Hoyer, 2020). In spite of this remarkable track record, the full potential of synergetic gain from a systematic coordination of research at the clinics had until recently not yet been sufficiently exploited. The scientific evaluation of treatment data is particularly difficult for clinics with a smaller number of cases: Patients and therapists often invest time and effort to answer questions about symptoms, the course of therapy or therapeutic relationships without sufficiently large samples for quantitative analysis. Up to now, the combination of the collected data with other clinics has been an exception that was limited to individual multicenter research projects (e.g., Gloster et al., 2011; Hoyer et al., 2016; Lutz et al., 2009). Nonetheless, the chances of an aggregation of research data across clinics are manifold.

Research coordination would involve a standardization in diagnostic documentation, a standardized reporting system and consequently the possibility of aggregating data from several or all outpatient clinics. Proposals for practice research networks have already been discussed on various occasions (e.g., Borkovec et al., 2001; Castonguay, 2011). A collaborative approach offers a number of important advantages: With the aggregated basic data, research with a large number of cases can be carried out in a short time. If necessary, comparatively rare disorders or their variants (e.g., Skin Picking Disorder, Depersonalization/Derealization Disorder, Sexual Dysfunctions; Balon, 2017; Sierra & David, 2011; Velten et al., 2021) even those not yet explicitly defined in classification systems (e.g. Facebook Addiction Disorder; Brailovskaia et al., 2018, 2019) can be investigated. In the case of more frequent disorders, the high number of cases allows subgroup comparisons and valid benchmark analyses to be carried out. Current topics such as the investigation of therapist data, discontinuation rates, the hotly debated topic of failures and side effects (Jacobi et al., 2011), transgenerational psychotherapy effects (Schneider et al., 2013) or groundbreaking developments in basic research (such as in the area of therapygenetics; Coleman et al., 2017; Rayner et al., 2019; Roberts et al., 2017, 2019; Wannemüller et al., 2018a; Wannemüller et al., 2018b) could be addressed more quickly with highly visible studies based on large clinical data sets. Ultimately, the collaborative database provides a valuable starting point for applying for major projects.

In 2013, an initiative group began to lay the groundwork for the systematic coordination of research in the German university outpatient clinics for psychotherapy in order to create a nationwide research platform for clinical psychology and psychotherapy (German abbreviation “KODAP” for “Coordination of Data Acquisition at Research Clinics for Psychotherapy”). This platform will allow the aggregation and analysis of longitudinal treatment data – including patient, therapist, and treatment characteristics – across all participating clinics for adults, children and adolescents. The short-term goal of KODAP was to establish the feasibility of large-scale coordinated research. Medium to long-term goals of the project are the advancement of theory, practice, and dissemination of psychotherapy and clinical psychology. The present article describes the steps taken, the challenges that had to be overcome and four feasibility studies that were carried out.

## Overview of Feasibility Studies

Immediate goals of Study 1 (Hoyer et al., 2015) were (a) to gather information on the core characteristics of the clinics and on this basis (b) to develop proposals for better integration of research efforts. In order to estimate the size and clinical composition of potential populations for future studies the number of patients initiating treatment in the participating KODAP outpatient clinics in 2016 as well as their diagnoses and psychopathological complaints together with the database, research and administrative software used in the clinics were recorded. Immediate goals of Study 2 (Velten et al., 2017) were (a) to develop a comprehensive catalogue of the considerable logistical, technical and legal data protection challenges facing the planned research collaboration, (b) to use this to examine the workability of cross-clinic collection of patient, therapist and therapy data and (c) to plan the third and fourth pilot studies. Study 3 (Velten et al., 2018) and Study 4 (In-Albon et al., 2019) aimed (a) to actually aggregate patient data across a pilot sample of clinics (Study 3: adults, Study 4: children and adolescents) treated in 2016 and use this (b) to test all the processes necessary for data preparation, transmission and aggregation at the cooperation partners and the central coordination center. The focus was on the frequency distribution of treatment diagnoses to answer the following research questions: Which disorders are frequently treated, which are rarely? How high is the proportion of severely distressed patient groups with more than one disorder diagnosis, at least one personality disorder or severe symptoms?

### Study 1 (Hoyer et al., 2015)

#### Method

A complete list of outpatient clinics at German university departments of clinical psychology and psychotherapy for the psychotherapeutic treatment of adults, children and adolescents (referred to as “clinics” in the following) was compiled in 2014 (Hoyer et al., 2015). This yielded 53 institutions whose scientific and managing directors were contacted by e-mail in May 2014 with the request to complete a short survey form. A questionnaire was developed by the initiative group to record the characteristics of the clinics. It asked for the diagnostic instruments, disorder-specific and general clinical questionnaires, as well as the patient and therapist variables of interest. In addition, the type, strengths and weaknesses of the clinical, research and administrative software used was assessed by open questions. Finally, the clinics reported the annual number of pre and post therapy datasets of all patients (i.e., defined as any person for whom a patient file was created) treated in 2013. Case numbers for adults and children and adolescents were asked separately.

#### Results

All 53 clinics contacted provided data on their institution by November 2014 (100% response rate). Whereas some of the clinics were still in the planning or construction stage or could not provide reliable data on current patient numbers for technical reasons, 49 clinics were able to provide information on their annual number of patients. Estimates (some of the clinics were able to provide only approximate data) for patients treated in 2013 yielded 8200 pre- and 5400 post-therapy data records for adults, and 2400 pre- and 1100 post-therapy data records for children and adolescents.

There were clear overlaps in the methods used for the diagnosis of mental disorders as shown in Table 1. Given the large number of different mental disorders treated in the clinics, it is not surprising that more than 150 different disorder-specific instruments were identified by the survey.

**Table 1:** Diagnostic Assessments Utilized Routinely in Outpatient Clinics (Instruments Used by at Least 15% of Clinics).

Instrument	% of clinics using instrument
Instruments used for ICD/DSM diagnoses	
*Adults*	
Structured Clinical Interview for DSM-IV^a^, SCID	89.2
International Diagnostic Checklist^b^, IDCL	21.6
Diagnostic Interview for Mental Disorders^c^, DIPS	16.2
*Children and adolescents*	
Diagnostic Interview for Mental Disorders in Childhood and Adolescence^d^, Kinder-DIPS	85.7
General clinical instruments	
*Adults*	
Brief Symptom Inventory^e^, BSI	62.2
Symptom Checklist 90-Revised^f^, SCL 90-R	45.9
Inventory of Interpersonal Problems^g^, IIP	27.0
Clinical Global Impressions Scale^h^, CGI	24.3
*Children and adolescents*	
Child Behavior Checklist^i^, CBCL/6-18R	64.3
Youth Self-Report of the Child Behavior Checklist^i^, YSR/11-18R	57.1
Teacher Report Form^i^, TRF/6-18R	50.0
Inventory for the Assessment of Life Quality in Children and Adolescents^j^, ILK	42.9
Disorder-specific instruments	
*Adults*	
Beck Depression Inventory^k^, BDI I or BDI II	89.2
Body Sensations Questionnaire, Agoraphobic Cognitions Questionnaire, Mobility Inventory^l^	64.9
Screening for Somatoform Symptoms 2^m^, SOMS 2	56.8
Eating Disorder Inventory 2^n^, EDI 2	48.6
Social Interaction Anxiety Scale^o^, SIAS	48.6
Hamburg Obsessive/Compulsive Inventory^p^, HZI	45.9
Social Phobia-Scale^o^, SPS	43.2
Posttraumatic Stress Diagnostic Scale^q^, PSD	40.5
Impact of Event Scale^r^, IES	35.1
Eating Inventory^s^, FEV	29.7
Borderline-Symptom-List-23^t^, BSL-23	29.7
Yale Brown Obsessive Compulsive Scale^u^, Y-BOCS	27.0
*Children and adolescents*	
Children's Depression Inventory^v^, DIKJ	64.3
Fear Survey Schedule for Children – Revised^w^, PHOKI	57.1
Social Phobia and Anxiety Inventory for Children^x^, SPAIK	35.7
Anxiety Questionnaire for School Students^y^, AFS	35.7

^a^Wittchen et al., 1997. ^b^Hiller et al., 1997. ^c^Margraf et al., 2017; Schneider & Margraf, 2011. ^d^Margraf et al., 2017; Schneider et al., 2009. ^e^Derogatis & Spencer, 1993; Franke, 1997. ^f^Derogatis, 1992; Franke & Derogatis, 1995. ^g^Horowitz et al., 2000. ^h^Guy, 1976; Kadouri et al., 2007. ^i^Döpfner et al., 2014. ^j^Mattejat & Remschmidt, 2006. ^k^Hautzinger et al., 2000, 2009. ^l^Ehlers et al., 2001. ^m^Rief et al., 1997. ^n^Paul & Thiel, 2004. ^o^Stangier et al., 1999. ^p^Zaworka et al., 2003. ^q^Griesel et al., 2006. ^r^Maercker & Schützwohl, 1998. ^s^Pudel & Westenhöfer, 1989. ^t^Wolf et al., 2009. ^u^Hand & Büttner-Westphal, 1991. ^v^Stiensmeier-Pelster et al., 2014. ^w^Döpfner et al., 2006. ^x^Melfsen et al., 2001. ^y^Wieczerkowski et al., 1981.

The systematic collection of essential patient characteristics such as age, gender and diagnosis (see Table 2) is a standard in all participating clinics. In addition, most clinics also record level of education, marital status and the number of therapy sessions. The documentation of therapist characteristics is limited to therapist gender, age and training status in most clinics. A large number of different software programs for patient data maintenance, room planning and billing as well as other administrative purposes are used by the clinics. These include programs from commercial providers as well as individual database solutions created in-house. The three most frequently cited software tools were PsychoEQ (PsychoWare Software), AMBOS (Therapy Organization Software) and self-developed SPSS or Microsoft Excel databases. The most frequently named strengths of the respective software solutions are their individual adaptability to the needs of the clinic, easy exportability of the data, simple operation and good support from the manufacturer. Frequently mentioned weaknesses of the programs are the susceptibility to errors, the limitation of data export only via employees of the manufacturer as well as the missing possibility to record specific variables such as therapist characteristics.

**Table 2:** Patient and Therapist Characteristics Reported in Feasibility Study 1

Variable	% of clinics giving information
Patient characteristics	
Age	100
Gender	100
Diagnosis (ICD-10)	100
Level of education	95.9
Marital status	93.9
Number of treatment sessions	93.9
Index diagnosis	89.8
Therapist characteristics	
Gender	77.6
Age	69.4
Training status (fully licensed vs. in training)	65.3

### Study 2 (Velten et al., 2017)

#### Method

The results of the first pilot study were evaluated by the initiative group^1^1C. Bennecke, M. Berking, J. Hoyer, T. In-Albon, T. Lincoln, W. Lutz, J. Margraf, A. Schlarb, H. Schöttke, U. Willutzki. in several face-to-face meetings as well as in telephone and Skype conferences in 2015 and 2016. Two subgroups dealt with the variables for adults and for children/adolescents, respectively. This led to the following structure of the catalogue of logistical, technical and legal data protection challenges facing the planned research collaboration: (1) organizational framework conditions, (2) cooperation agreement, (3) Steering Group, (4) coordination center, (5) initial set of variables to be collected for adults and for children and adolescents, (6) process to expand the dataset in the future, (7) data protection of transmitted information and ethical approval, (8) planning of the final feasibility study (Velten et al., 2017). For each of these sections specific recommendations were formulated on the basis of unanimous decisions. In addition, the procedures for patient informed consent and ethical approval of the project had to be developed.

#### Results

Based on the results of Study 1, the initiative group for the development of research cooperation derived recommendations regarding the catalogue of challenges for the cooperation project listed below. All recommendations were formulated on the basis of unanimous decisions by the initiative group.

##### (1) Organizational framework conditions

The planned research cooperation requires a solid organizational basis that must be supported by a legal entity. On 20 March 2017, *Unith.ev* began to serve as the organizing institution of the KODAP project. *Unith.ev* (the network of German university outpatient clinics for psychotherapy) is a registered non-profit association (the German “ev” stands for registered association, “unith” combines “university” and “therapy”). The sponsorship by a registered association clarifies the continued legal responsibility, and the non-profit character underlines the non-commercial character of its research, which serves the common good.

##### (2) Cooperation agreement

In order to legally secure the ambitious project, a cooperation agreement was drafted which regulates the rights and obligations of all participating clinics. It specifies the subject matter of the contract and provides the relevant information on the duration, confidentiality, liability and termination of membership in the project. In order to ensure the effective execution of the scientific and operational work of the research network, a steering group and a coordination center had to be established. Their respective tasks are also defined in the cooperation agreement (in German language, available from the first author on request).

##### (3) Steering group

The tasks of the steering group include the development, support and conception of KODAP's research activities. At present (mid-2020), the steering group consists of most members of the initiative group, which was formed in October 2013 at the annual meeting of German university professors of clinical psychology and psychotherapy. So far, the group met about three times a year, addressing the essential steps of the project, taking decisions by consensus. It currently consists of 8 members, representing 8 different universities. Rules of procedure were adopted in January 2017 to govern the rights and duties of the steering group (in German language, available from the first author on request) and contain guidelines for publications based on KODAP data.

##### (4) Coordination center

The main tasks of the coordination center are the collection, storage, quality control, aggregation and statistical analysis of the data obtained. The data sets which the participating clinics provide annually for the KODAP project are aggregated and stored in the coordination center. This task was taken over by the Mental Health Research and Treatment Center of Ruhr University Bochum. Regular reports, which serve to keep the partners continuously informed about the progress of work, are prepared by the coordination center. The rights and duties of the coordination center are set out in the cooperation agreement (in German language, available from the first author on request).

##### (5) Initial set of variables

The initial core data set defined is presented in Table 3.

**Table 3:** Initial Core Set of Variables to be Collected for Adults and for Children and Adolescents

Patient characteristics
*All*
Age (years)
Gender
Previous psychological or psychosocial treatments
Index and additional diagnoses (ICD-10, before and after therapy) based on structured or standardized clinical interviews
Level of education
Clinicians Global Impression Scale^a^, CGI
*Adults*
Marital status
Brief Symptom Inventory^b^, BSI or Symptom Checklist 90-Revised^c^, SCL 90-R
Beck Depression Inventory^d^, BDI I or BDI II
*Children and adolescents*
Child Behavior Checklist^e^, CBCL
Youth Self-Report of the Child Behavior Checklist^e^, YSR 11-18R
Psychosocial stressors (max. 5)
Living situation
Parent variables: BSI^b^ or SCL-90-R^c^, level of education, partnership status
Therapist characteristics
Gender
Age
Training status (fully licensed vs. still in training)
Treatment variables
Number of therapy sessions
Type of treatment performed
Current treatment status (ongoing, discontinued, regular termination)

^a^Guy, 1976; Kadouri et al., 2007. ^b^Derogatis & Spencer, 1993; Franke, 1997. ^c^Derogatis, 1992; Franke & Derogatis, 1995. ^d^Hautzinger et al., 2000, 2009. ^e^Döpfner et al., 2014.

The aim of assessing only a limited number of variables was to minimize the additional burden of data collection for KODAP and to allow clinics to continue using established assessments. Since the psychometric instruments are given before and after treatment, it is possible to evaluate therapy outcome.

All patient and therapist data are collected in pseudonymized form. Special consideration needs to be given to the problem of personal data, as is emphasized in Article 26 of the basic EU data protection regulation (see Regulation [EU] 2016/679; European Parliament and Council, 2016), which became effective in May 2018. KODAP follows the recommendations of a task force of the German Society of Psychology. As a consequence, the KODAP project does not collect data that are used in combination by a "person at his or her own discretion [...] to identify the natural person directly or indirectly" (Article 26). In order to ensure that individual patients - even those with rare disorders - cannot be identified on the basis of personal characteristics such as occupation or date of birth, only basic characteristics (level of education, age in years, gender, pre- and post-therapy diagnoses) are to be collected in the KODAP project. This procedure enables the storage of different data for a given patient over several years necessary for the longitudinal data collection, one of the central goals of KODAP. The same considerations also apply to the selection of therapist variables; therefore only information on age, gender and training status are recorded. With respect to treatment variables, the current treatment status (ongoing, completed or discontinued therapy), number of sessions and type of psychotherapeutic procedure are stored.

##### (6) Process to expand the dataset in the future

Since the success of KODAP essentially depends on smooth and reliable data collection and combination, only a manageable number of patient, therapist and therapy variables should be transmitted at the start of the project. However, a particular strength of a large-scale collaborative project is that it allows the investigation of rare disorders or therapy phenomena as well as new survey instruments. An extension of the initial data set is therefore planned for the future. It is relatively easy to extend the data set with instruments or variables, of which we know from Study 1 (Hoyer et al., 2015) that the majority of clinics already use them (e.g., SPS, SIAS, SOMS 2, EDI 2). In the long term, the survey can be expanded by follow-up data through multiple measurements across the course of therapy as well as freely available psychometric instruments. Similar to the British Improving Access to Psychological Therapies (IAPT) (Clark, 2018) program, KODAP will also serve to develop and establish public domain instruments. In addition, all participating project partners are free to propose additional time-limited research questions. If an additional variable that is relevant for many patients is specifically collected over a clearly defined period (e.g., 3 or 6 months) in all clinics, large, clinically well-documented samples can be obtained in a very short time.

##### (7) Data protection and ethical approval

As the variables to be collected in the clinic include sensitive treatment and health data special attention had to be given to data protection aspects in the run-up to the project as discussed in section (5) above. With regard to data transmission, various technical implementations were examined by the steering group. The solution needed to ensure longitudinal data collection, secure data transmission and storage, easy application by the clinic and low maintenance in the coordination center. In order not to delay the start of the project due to costly and time-consuming technology, we decided to merge the data records into one SPSS data record. A corresponding SPSS template (for adults or children and adolescents) is provided to all participating clinics at the start of the project, which will be sent back to the coordination center on encrypted data carriers at the end of the first project year. The data are stored in secured form on the server of the coordination center. In order to ensure that the transfer of patient data in KODAP is ethically acceptable, an informed consent form was developed, which has to be signed by the patients before the start of treatment (in German language, available from the first author on request). Before the start of the project, the ethics committee of the Faculty of Psychology at Ruhr University Bochum approved the project. The clinics are, however, free to additionally secure their participation in the project by submitting their own applications to their local ethics committees.

##### (8) Planning of the final feasibility studies

The first transmission of data, which form the basis for longitudinal analyses over several years, was planned to take place between the clinics and the coordination center in January 2019. At this point, the core data of those patients whose treatment started in 2018 were to be transmitted. Before this, however, it was planned to pilot the processes necessary for data preparation, transmission and aggregation at the cooperation partners and the coordination center. For this purpose, the clinics that joined the project by September 2017 provided the patients' core data sets from 2016 for two final (the third and fourth) feasibility studies. The benefits of these feasibility studies go far beyond the mere optimization of the project processes as descriptive statistics of patient data (e.g., distribution of diagnoses, age structure, type and number of co-morbidities and severity of treated disorders) are not yet available for German psychotherapy clinics.

### Study 3 (Velten et al., 2018)

#### Method

As of June 2018, 32 clinics from 15 locations had joined the KODAP project (26 for adults and 6 for children and adolescents). These were invited to contribute the initial core set for adult patients (see Table 3). All patients treated in the participating clinics in 2016 as well as their therapists were to be included, no other inclusion or exclusion criteria applied. A total of 16 clinics for adults were able to provide data sets (Velten et al., 2018). Reasons for non-participation were the lack of data due to the recent establishment of clinics and the missing approval by ethics committees for the transmission of data from 2016 because of a lack of coordinated consent forms. The participating clinics checked their internal data for completeness and compatibility and assessed the time and personnel required to process and transmit the data. In the coordination center data quality and ease of data transmission were tested. Faulty data points were reported back to the clinics. In addition, study protocols with precise information on all variables were sent to the clinics, which were to be returned to the coordination center together with the quantitative data set. A qualitative evaluation of the study protocols was used to check the variables for conclusiveness and to identify difficulties in data collection.

In order to prevent possible personal identification, some variables (e.g., occupation, exact time of treatment, transgenderness) were not collected. ICD-10 F diagnoses (Dilling, Mombour, Schmidt, & Weltgesundheitsorganisation, 2005; World Health Organization, 1993) at the beginning of treatment were recorded separately for the initial or index diagnosis (defined as the main reason for presentation) and for additional diagnoses. Reported diagnoses had to be derived from a standardized diagnostic tool or a structured interview according to ICD-10, DSM-IV or DSM-5. In addition to the patient, therapist, and therapy variables listed in Table 3, the average number of patients treated during the study period was computed.

#### Results

Of the 26 KODAP adult clinics, 16 clinics (61.5%) from ten locations (Humboldt-Universität zu Berlin, Freie Universität Berlin, Bochum, Dresden, Greifswald, Hamburg, Landau, Mainz, Trier, Osnabrück) provided data on 4504 individuals treated in 2016 (start of treatment could have been in 2016 or earlier). The number of records transmitted per clinic ranged from 24 to 756. The completeness and quality of the data (e.g. with regard to the coding of the response options) were checked in the clinics. With the support of the coordination center, all clinics were able to adapt their internal data collection in such a way that all defined variables for the future longitudinal study could be transmitted in an adequate form. All participating clinics were able to provide the time and personnel resources needed for the preparation and transfer of the data records. All clinics transmitted the data sets to the coordination center in compliance with data protection regulations (Velten et al., 2017).

##### Patient sociodemographic

The majority of the persons treated (mean age = 37.87; *SD* = 13.47; Range = 15-86 years) were female (*n* = 2937, 65.3%) and currently in a partnership (*n* = 2383, 67.5%). Marital status was reported as 49.4% (*n* = 1777) single, 29.4% (*n* = 1058) married and 9.2% (*n* = 332) divorced. The highest school degree attained was the German “Abitur” (equivalent to A-level or International Baccalaureate Diploma) for 48.2% (*n* = 1518), intermediate school certificate (German “Mittlere Reife”) for 29.4% (*n* = 926) and basic school certificate (German “Hauptschulabschluss”) for 18.1% (*n* = 570). At the start of treatment, 68.7% (*n* = 803) of the patients were able to work. In addition to the 18.6% (*n* = 217) disabled patients, 5.5% (*n* = 64) received a retirement pension and 3.1% (*n* = 36) an invalidity pension.

##### Patient diagnoses

**Figure 1:**
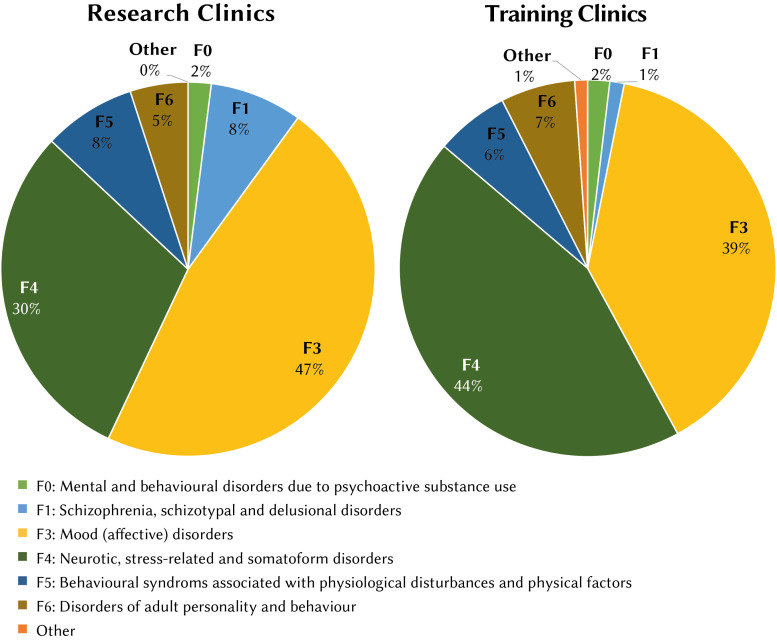
Proportion of Patients Treated in Research and Training Clinics by Index Diagnosis (ICD-10)

Nearly all clinics stated that the diagnosis at the beginning of treatment was confirmed by structured or standardized interview procedures. Only one outpatient clinic reported that an interview was not always used. A total of 7947 diagnoses were assigned to 4266 patients. Neurotic, stress and somatoform disorders (F4) were the most common category, followed by affective disorders (F3). A recurrent depressive disorder, currently a moderate episode (F33.1), was diagnosed 844 times, making it the most common disorder. With 651 and 539 assigned diagnoses, social phobia and the moderate depressive episode were the second and third most common disorders. Personality and behavioral disorders were diagnosed a total of 563 times. At least one personality disorder (F60 or F61) was present in 10.8% of all patients. The distribution of index diagnoses, which were defined as treatment causes in this study, differed from that of the overall distribution of all diagnoses assigned. Although F4 diagnoses were the most frequently assigned, affective disorders (F3) were by far the most frequent index diagnoses with 39.4% (*n* = 1682). Phobias (F40.-) and other anxiety disorders (F41.-) accounted for 14.2% (*n* = 607) of the initial diagnoses. Also frequently given were index diagnoses in the area of somatoform disorders (F45.-) with 5.5% (*n* = 233), post-traumatic stress disorder (F43.1) with 4.5% (*n* = 190), adaptation disorders (F43.2) with 4.5% (*n* = 190), eating disorders (F50.-) with 4.4% (*n* = 186) and emotionally unstable personality disorder: borderline type (F60.31) with 2.6% (*n* = 113). However, patients with bipolar affective disorders (*n* = 42; 0.9%), schizophrenia (*n* = 44; 1.0%) and sexual dysfunction (*n* = 8; 0.2%) as index diagnoses were rarely treated. The average number of diagnoses given was 1.84 (*SD* = 0.99, range = 0-7). Thus, multimorbidity was found in the majority of cases. 43.1% (*n* = 1865) had only one diagnosis, 33.4% (*n* = 1448) had two and 21.6% (*n* = 942) had three or more. Only 1.7% (*n* = 74) had no diagnosis at the start of treatment or no diagnosis was recorded in the system. The most frequent comorbidity pattern was the co-occurrence of affective disorders (F3) and neurotic, stress and somatoform disorders (F4). For example, 581 patients (13.7%) with F4 index diagnosis had an additional F3 diagnosis. The reverse pattern, F3 as first diagnosis and F4 as second and/or third diagnosis, applied to 546 patients (12.8%). Figure 1 shows the proportion of patients treated in research and training clinics by index diagnosis (ICD-10).

Table S1 in the Supplementary Materials shows the 50 most frequently assigned diagnoses, broken down by main disorder categories. Table S2 in the Supplementary Materials shows the 50 most frequently assigned index diagnoses, which were defined as treatment causes in this study. Table S3 in the Supplementary Materials shows the most frequent diagnostic combinations or comorbidity patterns after ICD-10-F disorder sections.

##### Patient psychopathological symptoms

Four clinics (*n* = 844 patients) provided data on the severity of the impairment at the start of therapy as assessed by the CGI. According to their therapists, 0.1% of the patients were not ill at all, 1.1% were borderline cases of mental disorder, 5.9% were only mildly ill, 28.9% were moderately ill, 49.8% were markedly ill, 12.1% were severely ill and 0.7% were among the most extremely ill patients. Table 4 shows the BSI and BDI values at the start of therapy. At the start of treatment, clinically relevant elevated BSI values (GSI > 0.61) were present in 76% (*n* = 2823), clinically significant BDI values (total values in BDI-I or BDI-II > 14) in 70% (*n* = 2298) of the treated persons. Severe depression symptoms (total values in BDI-I or BDI-II > 29) were reported by 24.3% (*n* = 797) of patients at the start of treatment.

**Table 4:** Level of Patients´ Psychopathological Symptoms at the Beginning of Treatment

Instrument	*n*	*M*	*SD*
Brief Symptom Inventory^a^, BSI	3753	0.89	0.77
Somatization	3757	1.47	0.87
Obsession-Compulsion	3758	1.44	1.00
Interpersonal Sensitivity	3760	1.36	0.93
Depression	3754	1.14	0.83
Anxiety	3760	0.96	0.76
Hostility	3756	0.85	0.88
Phobic anxiety	3760	1.10	0.88
Paranoid ideation	3756	0.92	0.77
Psychoticism	3763	1.12	0.67
Beck Depression Inventory^b^, BDI			
BDI-I	642	18.47	10.10
BDI-II	640	22.08	11.73

^a^Derogatis & Spencer, 1993; Franke, 1997. ^b^Hautzinger et al., 2000, 2009.

##### Psychotherapeutic treatments

In accordance with German psychotherapy regulations, a limited number of sessions are reserved for diagnostic procedures including case history and indicative decisions (so called probatory sessions). An average of 4.77 probatory sessions (*SD* = 0.85; range = 0-13) were performed. An outlier analysis showed only 1.5% of the treatments involved more than five probatory sessions. The number of regular therapy sessions after the probatory sessions was 35.01 (*SD* = 22.28, range = 0-117). While 42.7% (*n* = 1371) of the therapies were terminated consensually by patient and therapist (mean duration 43.09 therapy sessions, *SD* = 17.09), 23.3% (*n* = 748) were still ongoing at the time of data retrieval and 32.9% (*n* = 1057) of patients had dropped out of treatment (mean duration 23.8 sessions, *SD* = 22.04). In all cases, cognitive behavior therapy was used as therapeutic procedure. In the vast majority, only individual therapy sessions took place (90.9%, *n* = 2683), combined individual and group therapy were applied in 9.0% (*n* = 284) of the treatments.

##### Therapists

A total of 675 persons (mean age = 30.91 years, *SD* = 5.82, range = 22-58) were involved as therapists. Most therapists were female (*n* = 502, 83.3%) and the majority (*n* = 427, 70.6%) in advanced psychotherapy training (not licensed yet). On average, therapists treated 6.67 patients (*SD* = 5.75, range = 1-54) during the study period. An average of 5.19 (*SD* = 6.94, range = 1-43) patients per therapist were treated in the research clinics and 6.80 (*SD* = 5.29, range = 1-54) patients per therapist in the training clinics. An outlier analysis showed that 95% of therapists were responsible for less than 17 patients.

### Study 4 (In-Albon et al., 2019)

#### Method

This study characterized the patient population treated in 2016 in seven university outpatient psychotherapy clinics for children and adolescents (In-Albon et al., 2019). These submitted the initial core data set for children and adolescent patients (see Table 3). Completeness and quality of the data were checked in the clinics as well as in the coordination center as described in Study 3. Descriptive data on the diagnoses and comorbidity patterns of the patient population as well as sociodemographic information of their parents and therapists were analyzed. For the CBCL/6-18R and YSR/11-18R, *t*-values adapted for age and gender for a total, an externalizing and an internalizing score are reported.

#### Results

Study 4 characterized the patient population treated in 2016 in seven university outpatient psychotherapy clinics for children and adolescents. For the year 2016, data from 568 children and adolescents between 3 and 20 years of age (*M* = 11.89, *SD* = 3.68; 46.6% female) were available. The most frequent diagnoses were anxiety disorders (F40, F41, F93; *n* = 317, 35.30%) followed by attention-deficit hyperactivity disorders and conduct disorders (F90, F91, F92; *n* = 195, 21.71%). In 45.6% of the patients, there was at least one additional comorbid diagnosis. The mean *t-*value of the CBCL/6-18R (mother reports) was 67.60 (*SD* = 9.94) for the total score, 67.03 (*SD* = 10.70) for internalizing problems, and 61.84 (*SD* = 12.01) for externalizing problems. The mean *t*-value of the YSR/11-18R was 61.35 (*SD* = 10.23) for the total score, 63.43 (*SD* = 12.75) for internalizing problems, and 54.88 (*SD* = 9.53) for externalizing problems. All of these are above the clinical cut-off (*t* > 60; based on German norms; Döpfner et al., 2014). Therapist CGI severity scores classified the vast majority of patients as mentally ill (15.1% mildly, 46.6% moderately, 28.8% markedly, and 5.5% severely) and only few patients as not at all (1.4%) or borderline mentally ill (2.7%). Of the 126 therapists (83.1% female, mean 29.76 years, *SD* = 5.04), the majority (78.9%) were still in psychotherapy training (not licensed yet). Each therapist was responsible for a mean of 4.51 patients (range 1-13). Cognitive behavior therapy was used for all patients, and almost all treatments (99.3%) were conducted in an individual setting (combination of individual and group setting in 0.8%). An average of 6.93 probatory sessions (*SD* = 1.59, range 1-13) were performed. Most of the treatments (52.3%) had not yet been terminated. Overall, this study indicated the feasibility of consolidating and evaluating research data across university outpatient psychotherapy clinics for children and adolescents.

## Discussion

While other fields of research, such as physics, astronomy and genetics, have been practicing collaborative research on a large scale for some time, their value in the field of psychotherapy and mental health has only been increasingly recognized in recent years (Margraf, 2015). With the establishment of university outpatient clinics at departments of Clinical Psychology and Psychotherapy in Germany in 1999, a unique infrastructure for research, training and clinical care became available, offering opportunities for a collaborative approach. Since 2013, a steering committee works towards a systematic coordination across clinics in order to create a nationwide research platform. This platform will allow to aggregate and analyze longitudinal treatment data for adults, children and adolescents across all participating clinics and thereby contribute to the advancement of theory, practice and dissemination of psychotherapy and mental health research.

The feasibility of large-scale coordinated research was investigated in a series of four descriptive studies. An initial survey with 100% response rate (Study 1) in 2014 identified the most relevant features of the then 53 clinics and led to recommendations for improved integration of data collection. Already in 2014, the annual number of patients reported by the clinics surpassed 10,000 children, adolescents, and adults, with a strongly growing trend. Based on these results, we defined a catalogue of challenges facing the planned research collaboration and gave unanimously derived recommendations (Study 2). Study 3 collected data on 4,504 patients from 16 clinics treated in 2016 allowing for the first time to systematically describe patients, therapists and treatments available for collaborative research in the German psychotherapy outpatient clinic network. Finally, Study 4 analyzed data of 568 child and adolescent patients from seven clinics starting treatment 2016 providing the first description of this patient population within KODAP.

### Adult Patients

Diagnoses are based on evaluated, structured or standardized interviews whose validity and reliability exceed clinical judgment and other non-standardized diagnostic procedures (Margraf et al., 2017). The most frequently treated diagnostic groups in the KODAP clinics in 2016 were neurotic, stress and somatoform disorders (F4) and affective disorders (F3), the latter also yielding the most frequent index diagnoses and cause of treatment. This is in line with previous studies of psychotherapy outpatient clinics in Germany and England (Clark, 2018; Jacobi et al., 2011; Richter et al., 2013; Victor et al., 2018). The majority of KODAP patients (55%) had several mental disorders at the start of treatment. This is more than previously reported in non-university clinics (Victor et al., 2018), individual university clinics (Peikert et al., 2014; Richter et al., 2013) or routine care by practicing psychotherapists (Köck, 2012). While patients with almost all diagnoses and degrees of severity are treated, severe disorders (e.g., severe depressive episode, borderline disorder, chronic pain disorders, post-traumatic stress disorder) are very frequent. In addition, a sub-sample of four clinics showed that almost two thirds of the patients were rated by their therapists as markedly, severely or extremely ill. The fact, however, that psychotic disorders accounted only for one percent of treatment reasons (34th rank) calls for an increased proportion of this patient group in outpatient training settings (Schlier et al., 2017). Further investigation of the 7% of patients labeled by their therapists as borderline or only mildly ill may help to determine whether these patients may not have been in need of psychotherapy or whether some patient characteristics (e.g., certain diagnoses or symptoms, age, gender) may result in therapists’ underestimation of patient distress. While patients on average had a high level of education, a lack of comparative values prevented a direct comparison with earlier studies. The results for age and gender as well as the BSI and BDI scores show that the patient population in KODAP clinics is largely comparable to other German outpatient clinics and routine care by fully licensed behavior therapists (Jacobi et al., 2011; Köck, 2012; Lutz et al., 2013; Richter et al., 2013; Victor et al., 2018).

### Child and Adolescent Patients

The most frequently assigned diagnoses were anxiety disorders and behavioral disorders. This is in line with epidemiological studies, e.g. a meta-analysis (Polanczyk et al., 2015) indicating a prevalence rate of 6.5% for anxiety disorders, 5.7% for disruptive disorders, and 3.4% for ADHD. As in the adult clinics, the diagnoses are based on validated structured clinical interviews. The results of the questionnaires CBCL/6-18R and YSR/11-18R are comparable with a clinical control group of an outpatient sample in a child and adolescent psychiatric clinic (Walter et al., 2018). The categorical and dimensional diagnostic assessments as well as the comorbidity rate of almost 50% underline the clinical severity and the breadth of the problems treated in the participating child and adolescent clinics. The age range of 3 to 20 years reflects the legal restrictions for child and adolescent psychotherapists in Germany who may treat patients up to the age of 21. In contrast to the adult patient samples where roughly two thirds of the patients were female, girls and boys were equally distributed in the child and adolescent clinics.

### Therapists

The high proportion of female therapists (83%) is comparable with that of non-university training institutes (Victor et al., 2018) and somewhat higher than for practicing fully licensed psychotherapists in Germany (74.4%), or psychologist in the USA (73%) (APA Center for Workspace Studies, 2015). This reflects an ongoing international trend toward more women entering psychotherapy training and practice (APA Center for Workspace Studies, 2015). Because most of the reported treatments took place in training clinics, the majority of the therapists were not yet fully licensed. The fact that therapists treated an average of seven patients in training clinics during the study period underlines the intensity and structure of psychotherapy training in the participating clinics. Variability in number of patients treated per therapist in our data reflects the different training models (part-time vs. full-time training).

### Treatments

With an average of 43 treatment sessions for adults and 36 sessions for children and adolescents (regularly terminated therapies), the length of treatment is identical to that reported in other German outpatient clinics (Victor et al., 2018). This duration, however, is higher than internationally reported as the optimal dose for routinely delivered psychological therapies (Robinson et al., 2020). Patients dropped out in about one third of the treatments. Although this figure appears high, these values are comparable with termination rates reported in similar treatment settings (Hiller et al., 2009). In order to record the proportion of quality-relevant (e.g. low therapeutic success) in comparison to non-quality-relevant drop-outs (e.g., change of residence, low level of suffering), the reasons for early termination or non-execution of approved sessions should be systematically and uniformly documented in the future.

### Limitations

Although a large number of the clinics in question have already joined the KODAP project and more than half of the current member clinics contributed data to the last two feasibility studies, it is unclear to what extent the clinics included in this study are representative of all German university outpatient clinics for psychotherapy. Causes for non-participation of KODAP clinics in this study or reasons for missing variables in the transmitted data sets were not systematically documented. A more detailed, quantitative analysis of feasibility aspects related to data processing in clinics was therefore not possible. In addition, this study did not examine the extent to which clinics differ in terms of process and structural quality. Due to ethical and data protection considerations, only a limited number of personal variables of patients and therapists can be evaluated across clinics. A detailed analysis of the influence of specific personal variables, such as occupation or place of residence, is therefore not possible. Instead, this study deliberately focuses on a description of the patient population and treatment diagnoses at the beginning of treatment. The majority of clinics use the BDI-II, while two clinics still use the BDI-I. The comparability of the pre-treatment depression values across clinics with different BDI versions is therefore limited. Since the primary focus of this study was the estimation of feasibility aspects, the clinics were free to decide whether this first data transmission included variables already collected at the end of therapy. The analysis of treatment outcomes is planned for the longitudinal data collection that has been ongoing since the beginning of 2018.

### Opportunities and Challenges

The network provides a distinctive, unprecedented infrastructure for research, training and clinical care in psychotherapy and mental health. Clinical research designs, field experiments, and multicentric randomized controlled trials can be implemented rapidly and with large samples (e.g., 20 clinics per condition, inclusion of 1,000-5,000 patients), hence systematically solving typical problems such as recruitment issues, the lack of standardized assessments, and replicability.

Challenges for the collaborative project include expanding the core data set (e.g., behavioral data, social and biological variables), agreeing on new questions (e.g., long-term follow-up, systematic causality testing of predictors with experimental designs), and last but not least, full-cost funding of the joint research. A transfer of the network into a national structure would be desirable; a first application for consideration in the planned future National Research Center for Mental Health has already been submitted. The proof of a successfully established patient flow and the smooth realization of the cooperation will also improve the chances of success for acquisition of further third-party funding.

### Conclusions

Despite different data formats, data transfer and aggregation proved feasible. Affective, neurotic, stress, and somatoform disorders accounted for most of the diagnoses within the adult patients and anxiety and behavioral disorders within the child and adolescent patients. In both groups, comorbidity was the rule rather than the exception. Overcoming legal, methodological, and technical challenges, a common core assessment battery was developed and data collection for KODAP started in 2018. As of today, 42 clinics have joined and 30 already have provided data. The compilation of selected core data from the participating clinics makes it possible to answer important scientific and technical questions. These include but are not limited to the provision of normative data on patient, therapist, parents (for the child sample) and treatment characteristics, the interactions of such variables (e.g., success in specific subgroups, interaction of patient and therapist characteristics), treatment outcomes under routine conditions, dropout rates as well as failures and side effects in therapy, rare disorders, subgroup analyses of frequent disorders, special comorbidity patterns, specific age groups (e.g., preschool age, primary school or adolescent age; older patients) and high-powered studies for the development of new instruments and treatments. The first steps of KODAP reported here show that research collaboration across university outpatient clinics is feasible, provided that clinics invest time and effort for data collection, data checking and data transfer. Fulfilling the need for stronger cumulative and cooperative research in psychotherapy and related fields will contribute to better knowledge about mental health, a core challenge to modern societies.

## Supplementary Materials

The Supplementary Materials include three tables listing the diagnoses of patients in Study 3 (for access see Index of Supplementary Materials below).

10.23668/psycharchives.4459 Supplement 1 Supplementary materials to "The cooperative revolution reaches clinical psychology and psychotherapy: An example from Germany" [Additional information]

MargrafJ.
HoyerJ.
FydrichT.
In-AlbonT.
LincolnT.

LutzW.
VeltenJ.
 (2021). Supplementary materials to "The
cooperative revolution reaches clinical psychology and psychotherapy: An
example from Germany"
[Additional information].
PsychOpen. 10.23668/psycharchives.4559PMC966712036397785

## Author Note

All authors except for the first, second, and last are listed in alphabetical order.
