# Supplementary material for: The Cooperative Revolution Reaches Clinical Psychology and Psychotherapy: An Example From Germany
Source: Clin Psychol Eur. 2021 Mar 10;3(1):e4459. doi: 10.32872/cpe.4459 (PMC9667120; doi:10.32872/cpe.4459)
Supplement: Supplement 1 [file cpe-03-4459-s1.pdf]

Supplemental material

The cooperative revolution reaches Clinical Psychology and Psychotherapy: An example from Germany

Jürgen Margraf[1]*, Jürgen Hoyer[2,] Thomas Fydrich[3], Tina In-Albon[4], Tania Lincoln[5], Wolfgang Lutz[6], Angelika Schlarb[7], Henning Schöttke[8], Ulrike Willutzki[9], Julia Velten[1]

[1] Mental Health Treatment and Research Center, Ruhr University Bochum, Bochum, Germany

[2] Clinical Psychology and Psychotherapy, Technical University of Dresden, Germany
[3] Department of Psychology, Humboldt-Universität zu Berlin, Germany
[4] Clinical Child and Adolescent Psychology and Psychotherapy, University of Koblenz-Landau, Germany
[5] Clinical Psychology and Psychotherapy, Universität Hamburg, Germany
[6] Clinical Psychology and Psychotherapy, Trier University, Germany
[7] Clinic Psychology and Psychotherapy of children and adolescents, Bielefeld University, Germany
[8] Clinical Psychology and Psychotherapy, Osnabrück University, Germany
[9] Clinical Psychology and Psychotherapy, University Witten/Herdecke, Germany

Supplementary Table 1

Frequencies of assigned disorder diagnoses divided according to disorder categories

Disorder category	Disorder or disorder category	ICD-10 Code	Number of assigned diagnoses	Frequency range (1 = most frequent diagnosis)
Mental and behavioral disorders due to psychoactive substance use	Mental and behavioral disorders due to use of alcohol: Harmful use	F10.1	86	27
	Mental and behavioral disorders due to use of alcohol: Dependence syndrome	F10.2	113	21
	Mental and behavioral disorders due to use of cannabinoids: Dependence syndrome	F12.2	38	45
	Mental and behavioral disorders due to multiple drug use and use of other psychoactive substances	F19.X	31	47
	Sum		268	
Schizophrenia, schizotypal and delusional disorders	Schizophrenia	F20.X	66	34
Mood (affective) disorders	Bipolar affective disorder	F31.X	55	38
	Depressive episode	F32.-	95	25
	Mild depressive episode	F32.0	184	11
	Moderate depressive episode	F32.1	539	3
	Severe depressive episode without psychotic symptoms	F32.2	98	24
	Other depressive episodes; Depressive episode, unspecified	F32.8; F32.9	42	41
	Recurrent depressive disorder	F33.-	173	15
	Recurrent depressive disorder, current episode mild	F33.0	221	9
	Recurrent depressive disorder, current episode moderate	F33.1	844	1
	Recurrent depressive disorder, current episode severe without psychotic symptoms	F33.2	150	16
	Recurrent depressive disorder, currently in remission	F33.4	179	12

	Dysthymia		F34.1	280	6
		Sum		2860	
Neurotic, stress-related and somatoform disorders	Phobic anxiety disorders	Agoraphobia; Agoraphobia without panic disorder	F40.0; F40.00	83	28
		Agoraphobia with panic disorder	F40.01	417	4
		Social phobia	F40.1	651	2
		Specific (isolated) phobias	F40.2	255	7
	Other anxiety disorders	Panic disorder [episodic paroxysmal anxiety]	F41.0	176	13
		Generalized anxiety disorder	F41.1	176	13
	Obsessive-compulsive disorder	Obsessive-compulsive disorder	F42.-	31	47
		Obsessive-compulsive disorder: Predominantly obsessional thoughts or ruminations	F42.0	41	44
		Obsessive-compulsive disorder: Predominantly compulsive acts [obsessional rituals]	F42.1	73	31
		Obsessive-compulsive disorder: Mixed obsessional thoughts and acts	F42.2	123	19
	Reaction to severe stress, and adjustment disorders	Post-traumatic stress disorder	F43.1	390	5
		Adjustment disorders	F43.2	251	8
		Reaction to severe stress, unspecified	F43.9	68	33
	Somatoform disorders	Somatoform disorders	F45.0	42	41
		Undifferentiated somatoform disorder	F45.1	71	32
		Hypochondriacal disorders	F45.2	116	20
		Somatoform autonomic dysfunction	F45.3X	31	47
		Pain disorders related to psychological factors	F45.4	66	34
		Pain disorder exclusively related to psychological factors	F45.40	50	39
		Pain disorder with related psychological factors	F45.41	105	22
		Sum		3216	
Behavioral syndromes associated with	Eating disorders	Anorexia nervosa; Atypical anorexia nervosa	F50.0, F50.1	76	30
		Bulimia nervosa, Atypical bulimia nervosa	F50.2, F50.3	124	18
		Eating disorder, unspecified	F50.9	128	17

physiological disturbances and physical factors		Nonorganic sleep disorders	F51.X	29	50
		Sexual dysfunction, not caused by organic disorder or disease	F52.X	29	50
		Psychological and behavioral factors associated with disorders or diseases classified elsewhere	F54	102	23
		Sum		488	
Disorders of adult personality and behavior	Specific personality disorders	Other personality disorders	F60.0; F60.1; F60.2; F60.4; F60.7; F60.8; F60.9	78	29
		Emotionally unstable personality disorder: Borderline type	F60.31	195	10
		Emotionally unstable personality disorder: Impulsive type	F60.3X	42	41
		Anankastic personality disorder	F60.5	32	46
		Anxious [avoidant] personality disorder	F60.6	94	26
		Mixed and other personality disorders	F61.X	65	36
		Habit and impulse disorders	F63.X	57	37
		Sum		563	
Behavioral and emotional disorders with onset usually occurring in childhood and adolescence	Hyperkinetic disorder		F90.X	50	39

Table 2
50 most frequent Index diagnoses

	ICD-10 Code	Disorder	Frequency	Valid percentages
1	F33.1	Recurrent depressive disorder, current episode moderate	529	12.4
2	F32.1	Moderate depressive episode	363	8.5
3	F40.1	Social phobia	301	7.1
4	F40.01	Agoraphobia with panic disorder	248	5.8
5	F43.1	Post-traumatic stress disorder	190	4.5
6	F43.2	Adjustment disorders	141	3.3
7	F33.0	Recurrent depressive disorder, current episode mild	124	2.9
8	F34.1	Dysthymia	114	2.7
9	F60.31	Emotionally unstable personality disorder: Borderline type	113	2.6
10	F33.	Recurrent depressive disorder	112	2.6
11	F41.0	Panic disorder [episodic paroxysmal anxiety]	109	2.6
12	F33.2	Recurrent depressive disorder, current episode severe without psychotic symptom	104	2.4
13	F40.2	Specific (isolated) phobias	103	2.4
14	F41.1	Generalized anxiety disorder	100	2.3
15	F32.0	Mild depressive episode	98	2.3
16	F42.2	Obsessive-compulsive disorder: Mixed obsessional thoughts and acts	75	1.8
17	F45.2	Hypochondriacal disorders	72	1.7
18	F32.2	Severe depressive episode without psychotic symptoms	70	1.6
19	F50.9	Eating disorder, unspecified	69	1.6
20	F32.	Major depressive disorder, single episode	63	1.5
21	F43.9	Reaction to severe stress, unspecified	60	1.4
22	F33.4	Recurrent depressive disorder, currently in remission	56	1.3
23	F54	Psychological and behavioral factors associated with disorders or diseases classified elsewhere	55	1.3
24	F50.2	Bulimia nervosa	54	1.3
25	F45.41	Pain disorder with related psychological factors	53	1.2
26	F20.0	Paranoid schizophrenia	44	1.0
27	F45.1	Undifferentiated somatoform disorder	40	0.9
28	F40.00	Agoraphobia, unspecified	33	0.8
29	F42.1	Obsessive-compulsive disorder: Predominantly compulsive acts [obsessional rituals]	33	0.8
30	F10.2	Mental and behavioral disorders due to use of alcohol: Dependence syndrome	32	0.7
31	F45.4	Persistent somatoform pain disorder	32	0.7
32	F50.0	Anorexia nervosa	30	0.7
33	F32.9	Depressive episode, unspecified	29	0.7
34	F43.22	Adjustment disorder with mixed anxiety and depressed mood	29	0.7
35	F60.6	Anxious [avoidant] personality disorder	26	0.6
36	F45.40	Pain disorder exclusively related to psychological factors	23	0.5
37	F43.20	Adjustment disorder, unspecified	20	0.5

38	F41.2	Mixed anxiety and depressive disorder	18	0.4
39	F50.3	Atypical bulimia nervosa	18	0.4
40	F61	Mixed and other personality disorders	18	0.4
41	F42.0	Obsessive-compulsive disorder: Predominantly obsessional thoughts or ruminations	17	0.4
42	F10.1	Mental and behavioral disorders due to use of alcohol: Harmful use	16	0.4
43	F90.0	Disturbance of activity and attention	16	0.4
44	F50.1	Atypical anorexia nervosa	15	0.4
45	F41.9	Anxiety disorder, unspecified	14	0.3
46	F33.9	Recurrent depressive disorder, unspecified	13	0.3
47	F42	Obsessive-compulsive disorder	13	0.3
48	F45.0	Somatoform disorders	13	0.3
49	F60.30	Emotionally unstable personality disorder: Impulsive type	12	0.3
50	F31.3	Bipolar affective disorder, current episode mild or moderate depression	11	0.3

Table 3

List of patients with diagnoses or combination of diagnoses (frequency > 19) sorted by ICD-F-sections (N = 4268)

First diagnosis	Second diagnosis	Third diagnosis	Frequency	Valid percentages
F1 Mental and behavioral disorders due to psychoactive substance use	Existing	Not itemized	58	1.4
F2 Schizophrenia, schizotypal and delusional disorders	None	None	60	1.4
	Existing	Not itemized	30	0.7
F3 Mood (affective) disorders	None		807	18.9
	Other (besides F1, F2, F4, F5, F6)	Not itemized	25	0.6
	F1 Mental and behavioral disorders due to psychoactive substance use	Not itemized	78	1.8
	F3 Mood (affective) disorders	None	66	1.5
	F3 Mood (affective) disorders	Other (besides F4)	27	0.6
	F3 Mood (affective) disorders	F4 Neurotic, stress-related and somatoform disorders	34	0.8
	F4 Neurotic, stress-related and somatoform disorders	None	311	7.3
	F4 Neurotic, stress-related and somatoform disorders	F1 Mental and behavioral disorders due to psychoactive substance use	26	0.6
	F4 Neurotic, stress-related and somatoform disorders	F4 Neurotic, stress-related and somatoform disorders	119	2.8
	F4 Neurotic, stress-related and somatoform disorders	F5 Behavioral syndromes associated with physiological disturbances and physical factors	20	0.5
	F4 Neurotic, stress-related and somatoform disorders	F6 Disorders of adult personality and behavior	36	0.8

	F5 Behavioral syndromes associated with physiological disturbances and physical factors	Not itemized	73	1.7
	F6 Disorders of adult personality and behavior	None	87	2.0
	F6 Disorders of adult personality and behavior	Existing	28	0.7
F4 Neurotic, stress-related and somatoform disorders	None		792	18.6
	F1 Mental and behavioral disorders due to psychoactive substance use	Not itemized	34	0.8
	F3 Mood (affective) disorders	None	375	8.8
	F3 Mood (affective) disorders	F3 Mood (affective) disorders	24	0.6
	F3 Mood (affective) disorders	F4 Neurotic, stress-related and somatoform disorders	89	2.1
	F3 Mood (affective) disorders	F5 Behavioral syndromes associated with physiological disturbances and physical factors	22	0.5
	F3 Mood (affective) disorders	F6 Disorders of adult personality and behavior	30	0.7
	F4 Neurotic, stress-related and somatoform disorders	None	154	3.6
	F4 Neurotic, stress-related and somatoform disorders	F3 Mood (affective) disorders	86	2.0
	F4 Neurotic, stress-related and somatoform disorders	F4 Neurotic, stress-related and somatoform disorders	41	1.0
	F4 Neurotic, stress-related and somatoform disorders	Other (besides F3 or F4)	23	0.5
	F5 Behavioral syndromes associated with physiological disturbances and physical factors	Not itemized	39	0.9

	F6 Disorders of adult personality and behavior	Not itemized	52	1.2
F5 Behavioral syndromes associated with physiological disturbances and physical factors	None		99	2.3
	Other (besides F3 or F4)	Not itemized	30	0.7
	F3 Mood (affective) disorders	Not itemized	91	2.1
	F4 Neurotic, stress-related and somatoform disorders	Not itemized	49	1.1
F6 Disorders of adult personality and behavior	None		70	1.6
	Other (besides F3 or F4)	Not itemized	34	0.8
	F3 Mood (affective) disorders	Not itemized	81	1.9
	F4 Neurotic, stress-related and somatoform disorders	Not itemized	47	1.1
Other diagnoses or mix of diagnoses			121	2.8
